# ﻿Molecular cytogenetic study on the scleractinian coral *Micromussaamakusensis* (Veron, 1990) (Hexacorallia, Anthozoa, Cnidaria): isolation of five fluorescence *in situ* hybridization markers

**DOI:** 10.3897/compcytogen.19.157310

**Published:** 2025-08-07

**Authors:** Analyn B. Baldove, Masumi Ito, Takahiro Taguchi, Takuma Mezaki, Hiroumi Saito, Sam Edward Manalili, Yuji Namura, Ivy Jamela Nieves-Brutas, Akira Tominaga, Satoshi Kubota

**Affiliations:** 1 Kuroshio Science Program, Graduate School of Integrated Arts and Sciences, Kochi University, Nankoku, Kochi 783-8505, Japan Kochi University Nankoku Japan; 2 Takara Bio Inc. 7-4-38 Noji-higashi, Kusatsu, Shiga 525-0058, Japan Takara Bio Inc. Kusatsu Japan; 3 Kuroshio Biological Research Foundation, Otsuki, Hata County, Kochi 788-0333, Japan Kuroshio Biological Research Foundation Kochi Japan

**Keywords:** Chromosome, DNA repeats, FISH, karyotype, rRNA

## Abstract

Scleractinian (stony) corals are foundational to reef ecosystems, yet their taxonomy remains unresolved due to morphological plasticity and limited cytogenetic data. This study presents the first molecular cytogenetic characterization of the scleractinian coral *Micromussaamakusensis* (Veron, 1990), employing fluorescence in situ hybridization (FISH) to isolate and map five DNA markers. Using the conventional Giemsa staining technique, *M.amakusensis* was found to have a diploid karyotype of 2n = 28, with a prominent homogeneously staining region (HSR) on the long arm of chromosome 12. Subsequently, five FISH markers designated as MA-H3 for *histone H3*, MA-5S for *5S rRNA*, MA-18/28S for *18S-28S rRNA*, MA-13C for centromeric region, and MA-TEL for telomeric region were cloned, sequenced, and mapped using FISH. FISH analysis revealed that the MA-H3 localized to the centromeric region of chromosome 1, MA-5S to the telomeric region of chromosome 4, MA-18/28S to the terminal region of chromosome 12 (coinciding with the HSR), MA-13C to the centromere of chromosome 13, and MA-TEL to multiple telomeric regions across several chromosomes. Sequence analysis confirmed marker identities and revealed conserved and novel repetitive elements. Furthermore, Genomic DNA hybridization (GDH) of whole-sperm DNA revealed signals collected at several telomeric regions, suggesting the presence of repetitive sequences. These cytogenetic markers enable the identification of at least 3 out of 14 chromosome pairs, allow for more precise karyotyping, and highlight chromosomal features that may help resolve coral classification and improve understanding of genome evolution. This research demonstrates the utility of molecular cytogenetics in stony coral systematics and provides new FISH markers for future comparative genomic studies.

## ﻿Introduction

Scleractinian (stony) corals are crucial for building coral reef ecosystems, providing habitats and resources for many marine species. These stony corals are found in shallow, sunlit waters across tropical and temperate regions worldwide, including Japan, which hosts a wide variety of both tropical and temperate coral species such as *Micromussaamakusensis* (Veron, 1990) (Nishihira and Veron 1995).

Despite their ecological importance and the existence of more than 800 described species (Veron 2000), the classification of these corals remains a major challenge. Traditional taxonomy based on skeletal features often conflicts with molecular analyses using ribosomal and mitochondrial DNA (Fukami et al. 2004; Ng et al. 2019), partly due to hybridization between closely related species and high morphological plasticity (Taguchi et al. 2017a; Kawakami et al. 2022). This taxonomic confusion complicates conservation, especially as climate change accelerates coral biodiversity loss, as observed in *Acropora* spp. (Faith and Richards 2012; Richards et al. 2016; Locatelli et al. 2024).

Classification using chromosomal information is also challenging because coral chromosomes are typically small with little variation in size and shape, rendering them morphologically undifferentiated (Taguchi et al. 2013, 2016, 2017a, 2017b; Vacarizas et al. 2021). This uniformity makes it difficult to reliably distinguish individual chromosomes and identify homologous pairs using conventional microscopic and staining techniques (Kenyon 1997; Flot et al. 2006; Taguchi et al. 2013, 2016, 2017a, 2017b; Anokhin et al. 2018), which often yield unclear banding patterns. The lack of clear chromosome markers further limits the identification of individual chromosomes and complicates accurate karyotyping (She et al. 2023).

To address these problems, new cytogenetic approaches are needed. Molecular cytogenetics, especially techniques such as fluorescence in situ hybridization (FISH), can reveal chromosome patterns, gene locations, and repetitive DNA elements that clarify evolutionary relationships and genome organization (Taguchi et al. 2013, 2014, 2016, 2017a, 2017b, 2020; Warchałowska-Śliwa et al. 2020; Vacarizas et al. 2021, 2023; Kawakami et al. 2022; Mezzasalma et al. 2022; Khensuwan et al. 2023). FISH markers provide effective tools for chromosome identification and have been used to study chromosomal changes, gene order, and chromosome evolution in various organisms (Levsky and Singer 2003; Dorritie et al. 2004; Taguchi et al. 2013, 2014, 2016, 2017a, 2017b, 2020; Tang et al. 2019; Micolino et al. 2020; Quercia et al. 2020; Vacarizas et al. 2021, 2023; Tao et al. 2021; Araya-Jaime et al. 2022; Kawakami et al. 2022). Despite its potential to resolve taxonomic conflicts, molecular cytogenetic research in corals has been narrowly applied. Pioneering studies have focused on families like Acroporidae and Merulinidae (Taguchi et al. 2013, 2014, 2016, 2017b, 2020; Vacarizas et al. 2021, 2023; Kawakami et al. 2022). The large and taxonomically complex Lobophylliidae family, a major reef component, lacks any molecular cytogenetic characterization to date, which hinders a comprehensive understanding of coral genome evolution.

Therefore, this study aims to advance the molecular cytogenetic characterization of scleractinian corals by isolating and sequencing five DNA clones that serve as FISH markers for *M.amakusensis*, a member of the family Lobophylliidae. By applying molecular cytogenetic techniques, we aim to establish a more accurate karyotype for this species, which will improve its classification and contribute to a better understanding of coral genome evolution.

## ﻿Material and methods

### ﻿Coral collection

**Figure 1.**
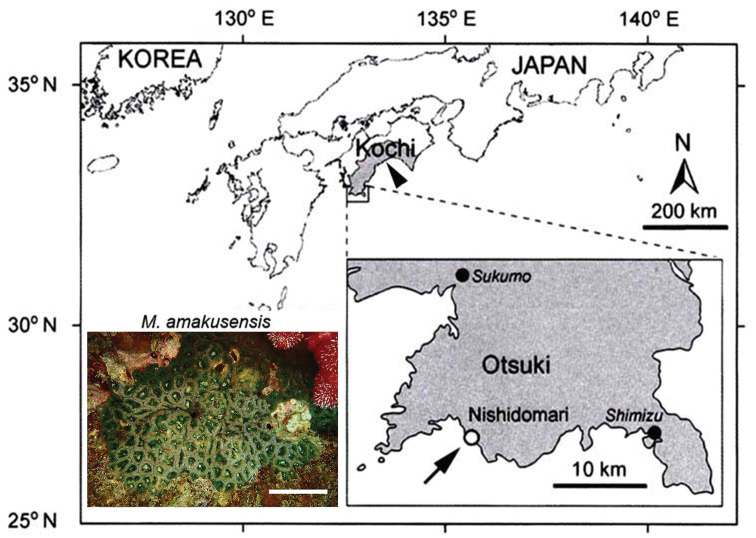
Collection site of *M.amakusensis*. Nishidomari (arrow) is located in the southwestern region of Kochi Prefecture (arrowhead) in Japan. The inset at the lower left shows the appearance of *M.amakusensis* in the sea. Scale bar: 5 cm.

*M.amakusensis* is simultaneously hermaphroditic, and spawning consists of the release of eggs and sperm packed together into discrete bundles from the mouths of fertile polyps. Gametes of *M.amakusensis* were collected at Nishidomari (32°46'N, 132°43'E) in Kochi Prefecture, Japan (Fig. 1).

The release of gamete bundles was observed between 21:00 and 21:45 on August 15, 2017, during which the bundles were collected using a bundle collector (plastic tent) placed over part of the colonies during spawning. We constructed the bundle collector by connecting a 15‐cm diameter funnel to a 50‐mL plastic test tube and deployed it at two separate sites to avoid fertilization between the same colony. The surfaces of the *M.amakusensis* colonies that were almost ready to release gamete bundles were tightly covered with the bundle collector. Gamete bundles collected from the two sites were transferred from the tubes to the same beaker (100 mL) and broken apart into eggs and sperm using gentle shaking. Eggs were collected using a pipette and were rinsed several times with 0.2-μm-filtered seawater (ADVANTEC cartridge filter; Advantec Toyo Corp., Tokyo, Japan) to remove external contaminants. Sperm were collected using centrifugation at 2,000 × g. Fertilized eggs were produced by mixing the sperm with the eggs collected from different *M.amakusensis* colonies. The fertilized eggs were subsequently rinsed with 0.2-μm-filtered seawater.

### ﻿Chromosome preparations

We previously reported a method for preparing coral chromosomes (Taguchi et al. 2014). Briefly, 9–12 h after artificial fertilization, embryos (blastula; the “prawn-chip” stage is used for scleractinian corals) were treated with 0.02% colchicine (dissolved in distilled water) (GIBCO; Invitrogen, Carlsbad, CA, USA) followed by a hypotonic solution to swell the cells and spread the chromosomes. The embryos were fixed using a freshly mixed solution of absolute methanol and glacial acetic acid (3:1). The fixed embryos were soaked in diethyl ether overnight to remove intracellular lipids and were then returned to the fixative. Twenty to thirty embryos were lysed by pipetting. Suspensions containing these small pieces of embryos were transferred into a 1.5 mL tube filled with fixative. The tube was centrifuged at 2,000 × *g* for 2 min, and the pellet was resuspended in 0.5 mL of fresh fixative. One drop containing fragments of embryos and isolated cells was placed on a clean slide and flame-dried to break the cell membranes and spread the chromosomes.

### ﻿Chromosome length measurements

FISH images were analyzed using Fiji software (https://imagej.net) to measure the chromosome dimensions. The absolute lengths of individual chromosomes and their long arms were determined by manually tracing using a freehand line tool. The total chromosomal length was obtained by summing the absolute lengths of all chromosomes within the karyotype. Relative chromosome lengths were calculated as the ratio of each chromosome’s length to the total chromosomal length. Additionally, centromere indices were determined as the ratio of the long arm length to the total chromosome length, following the method described by Levan et al. (1964).

### ﻿DNA extraction

Coral sperm DNA from *M.amakusensis* (approximately 1 × 10^6^) was extracted using the Wizard Genomic DNA Purification Kit (Promega Corporation, Madison, WI, USA), according to the manufacturer’s instructions.

### ﻿PCR and DNA cloning

FISH probes were generated by PCR using specific primers for *histone H3*, *5S rRNA*, *18S-28S rRNA*, and combinations of *5S rRNA* and *EF1-α* genes. MA-13C and MA-TEL markers used the same *5S rRNA* primer but different *EF1-α* primers. The MA-TEL probe was synthesized using the *5S rRNA* as the forward primer and the reverse primer derived from the reverse complement of the *EF1-α* primer, as shown in Table 1. The PCR reaction conditions were as follows: 40 cycles of 98 °C (10 seconds), 60 °C (30 seconds), and 72 °C (1 min) using a Minicycler (MJ Research, Waltham, MA, USA). The resulting PCR products were purified and ligated into a pGEM-T Easy-Vector (Promega, Corporation). The ligation products (10 ng) were used for transformation using competent cells (JM109; pGEM-T Easy Vector Systems; Promega KK, Japan). The cells were then spread on Luria-Broth (LB) plates containing 100 μg/mL ampicillin, 40 μg/mL 5-bromo-4-chloro-3-indolyl β-D-galactoside, and 0.05 mM isopropyl-β-D-1-thiogalactopyranoside. The plates were incubated for 15 h, and bacterial colonies were screened for positive insert using colony PCR. The positive colonies were grown to 15 mL test tubes containing 2.0 mL LB/ampicillin medium and incubated at 37 °C overnight. Plasmid DNA was extracted from these clones using a previously described method (Nucleospin; Macherey-Nagel GmbH & Co., Düren, Germany).

**Table 1.** Primer sets for the five fluorescence *in situ* hybridization markers.

	Name	Target genes	Primer sets: Sequence (5ʹ-3ʹ)	References
1	MA-H3	*Histone H3*	F- ATGGCTCGTACCAAGCAGACVGC	Huang et al. (2011)
			R- ATATCCTTRGGCATRATRGTGAC	Huang et al. (2011)
2	MA-5S	*5S rRNA*	F- GTTAAGCACCGTCAAGCCAGG	Stover and Steele (2001)
			R- CTTCCGTGATCGGACGAGAAC	Stover and Steele (2001)
3	MA-18/28S	*18S-28S rRNA*	F- TGGTTGATCCTGCCAGT	Wang et al. (2009)
			R- ATCCTTCNGCAGGTTCACC	Wang et al. (2009)
4	MA-13C	*5S rRNA*	F- CTTCCGTGATCGGACGAGAAC	Stover and Steele (2001)
		*EF1-α*	R- CCAATTTTGTAGACATCTTGAAG	Kawaida et al. (2010)
5	MA-TEL	*5S rRNA*	F- CTTCCGTGATCGGACGAGAAC	Stover and Steele (2001)
		*EF1-α*	R- ATTTACAAATGTGTGGTATCG	Kawaida et al. (2010)

### ﻿Probe preparation and FISH

Random primer labeling of the probe DNAs from the plasmid DNA was performed with fluorescein-12-dUTP (F-dUTP) or cyanine-3-dUTP (Cy3-dUTP) in accordance with the manufacturer’s protocol (Invitrogen). FISH analysis was performed as previously described (Taguchi et al. 2013). Briefly, metaphase preparations were denatured in 70% formamide/2× saline-sodium citrate at 70 °C for 2 min. Subsequently, 0.8 μL of probe was mixed with 10 μL of hybridization solution (H7782; Sigma, St Louis, MO, USA) and denatured at 80 °C for 10 min. Hybridization of the probes was performed at 37 °C for 12–18 h, followed by post-hybridization washes, counterstaining with 4ʹ,6-diamidino-2-phenylindole (DAPI), and visualization of the probes under a fluorescence microscope (BX50; Olympus, Tokyo, Japan).

### ﻿Genomic DNA hybridization (GDH) using M.amakusensis sperm DNA

*M.amakusensis* sperm DNA was labeled with Cy3-dUTP using a random priming kit (Invitrogen) according to the manufacturer’s instructions. One hundred nanograms (0.5 μL) of labeled DNA was dissolved in 10 μL of hybridization solution (H7782, Sigma), heat-denatured at 80 °C for 10 min, and then applied to slides containing denatured (at 70 °C for 2 min) metaphase spreads for hybridization. The chromosomes were counterstained with DAPI.

### ﻿DNA sequencing and Homology search

The inserts that were positive in FISH screening were sequenced using M13 forward and reverse primers with an ABI Prism BigDye Terminator Cycle Sequencing Ready Reaction Kit v2.0 and an ABI Prism 310 Genetic Analyzer (Applied Biosystems, Waltham, MA, USA). Computer-assisted DNA alignment and comparisons were performed using Mega BLAST (http://www.ddbj.nig.ac.jp).

### ﻿Image acquisition and Processing

The slides were examined under an Olympus BX-50 fluorescence microscope. Images of suitable metaphase spreads were acquired using an Olympus DP70 microscope workstation equipped with a cooled charge-coupled device and FISH analysis software. The Miller/filter units (Olympus) used to detect FITC, Cy-3, and DAPI, were U-NIBA, U-MWU, and U-MWIB, respectively.

## ﻿Results

### ﻿Karyotype of *M.amakusensis*

**Figure 2.**
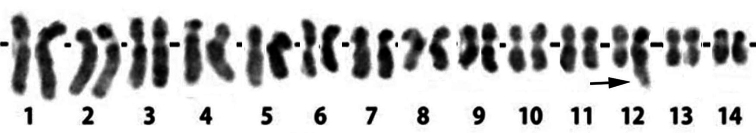
Giemsa stained karyogram of *M.amakusensis*. Note that chromosome 12 has an extra-pale long portion (HSR: arrow).

A survey of 50 metaphase spreads of *M.amakusensis* identified a complement of 28 chro­mosomes (2n = 28) (Fig. 2). Consequently, an HSR was identified in the long arm of chromosome 12 (Fig. 2). During the survey of Giemsa stained metaphase spreads, 31 spreads with HSR were found in the 60 cells examined (approximately 50%). As shown in Fig. 2, the chromosomes were arranged in decreasing order of length, from 1 to 14. While chromosome 12 was easily identified because of the presence of an HSR, the other chromosomes were not easily distinguished precisely because of the poor Giemsa staining pattern and similarities in chromosome length and centromere location. This karyotype consisted of seven submetacentric (1–7) and seven metacentric (8–14) chromosomes (Table 2).

**Table 2.** Relative lengths, centromere indices, and types of chromosome pairs.

Chromosome No.	Relative length	Centromere index	Chromosome type
1	10.99 ± 0.61	0.31 ± 0.03	sm*
2	9.73 ± 0.27	0.29 ± 0.05	sm
3	8.94 ± 0.42	0.30 ± 0.05	sm
4	8.35 ± 0.38	0.33 ± 0.08	sm
5	7.89 ± 0.34	0.35 ± 0.06	sm
6	7.46 ± 0.28	0.36 ± 0.06	sm
7	7.01 ± 0.30	0.38 ± 0.05	sm
8	6.77 ± 0.23	0.40 ± 0.06	m**
9	6.41 ± 0.27	0.43 ± 0.06	m
10	6.17 ± 0.27	0.39 ± 0.05	m
11	5.91 ± 0.30	0.40 ± 0.06	m
12	5.34 ± 0.40	0.41 ± 0.09	m
13	4.90 ± 0.38	0.46 ± 0.06	m
14	4.12 ± 0.48	0.51 ± 0.09	m

*sm: sub-metacentric; **m: metacentric. Means and standard deviations were obtained from 10 metaphase spreads. The types were categorized according to Levan et al. (1964).

### ﻿GDH with whole-sperm DNA

**Figure 3.**
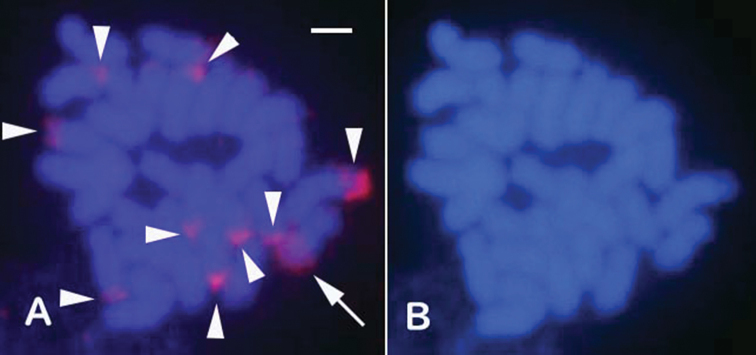
Fluorescence *in situ* hybridization images of GDH using whole-sperm DNA from *M.amakusensis*. **A.**GDH image using sperm DNA (genomic DNA) labelled with Cy3-dUTP. **B.** The corresponding 4ʹ,6-diamidino-2-phenylindole image. Note the red signals on the telomere regions of several chromosomes (arrowheads), with the largest reddish signal (arrow) indicating a homogeneously stained region (HSR).

Using GDH, 6 to 10 signals were observed in the telomere regions of the chromosomes (Fig. 3A). According to the analysis of several different metaphase spreads, FISH signals were mostly in the telomere regions, and the number of signals varied from metaphase to metaphase.

### ﻿Cloning, Sequence and FISH analysis of five markers

Five FISH markers were successfully isolated from *M.amakusensis* PCR products using cloning techniques. The cloned genes were sequenced and screened via FISH. Sequence homology analyses revealed that the MA-H3 FISH marker, composed of 282 bp (Suppl. material 1), closely matched the *histone H3* gene and its adjacent non-coding region in *M.amakusensis*. This FISH marker also showed high identity (99.25%) with the *Montastraeamultipunctata* histone H3 gene (95% query coverage), while other matches were to various *M.amakusensis histone H3* genes (98.84 to 99.22% identity, 91% query coverage). The MA-5S FISH marker comprised 920 bp (Suppl. material 2), corresponded to part of the *5S rRNA* gene, and was named MA-5S (*5S U2RNA*). This sequence had 90% homology with both *Actiniaequina 5S rRNA* (Actiniaria) and the *M.capitata* clone Mc0343 microsatellite sequence (Scleractinia). Additionally, the MA-5S FISH marker matched four regions on different chromosomes of the *Echinoporahorrida* genome (56 to 60% query cover, 75.28% to 76.46% identity) and nine predicted *5S rRNA* sequences from *Pocilloporaverrucosa* (4% query cover, 97.78% identity). The MA-18/28S FISH marker, 1,258 bp long (Suppl. material 3), and the top BLAST hit was *M.amakusensis* (94.86% identity, 80% query cover), with additional hits including *Phymastreamultipunctata* (95.34% identity, 64% query cover) and other *Micromussa* sequences. The MA-13C FISH marker, 111 bp in length (Suppl. material 4), produced hits exclusively with the *Echinoporahorrida* genome, matching chromosome 2 (89.23% identity, 57% query cover) and chromosome 16 (87.69% identity, 57% query cover). The MA-TEL FISH marker, 149 bp in length (Suppl. material 5) and yielded no significant sequence similarities. All sequence data are listed in Suppl. materials 1–5.

**Figure 4.**
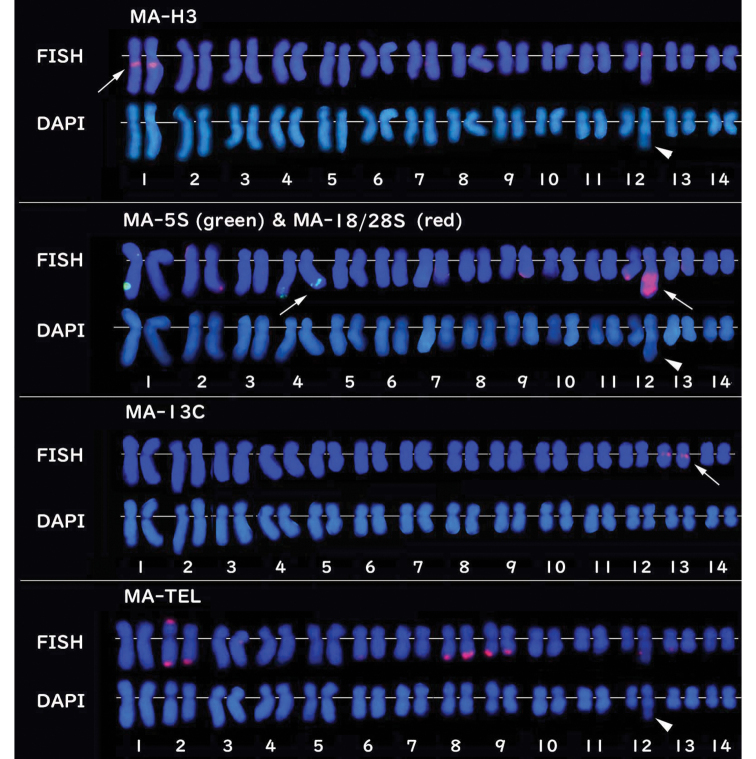
Karyotyped FISH images of five cloned probes. **A.** Red signals from MA-H3 (*histone H3*: arrows; upper row) and corresponding DAPI image (lower row). **B.** Dual-color FISH image (upper row) from MA-5S (arrows: green) and MA-18/28S (arrows: red) and corresponding DAPI image (lower row). **C.** MA-13C (arrows: red: upper row) and DAPI staining (lower row). **D.** Red signals from MA-TEL (upper row) and DAPI staining (lower row). The pale parts of long arms in **A, B** and **D** (arrowheads) in DAPI and one of the large red signal in B (arrow) indicate HSRs.

**Figure 5.**
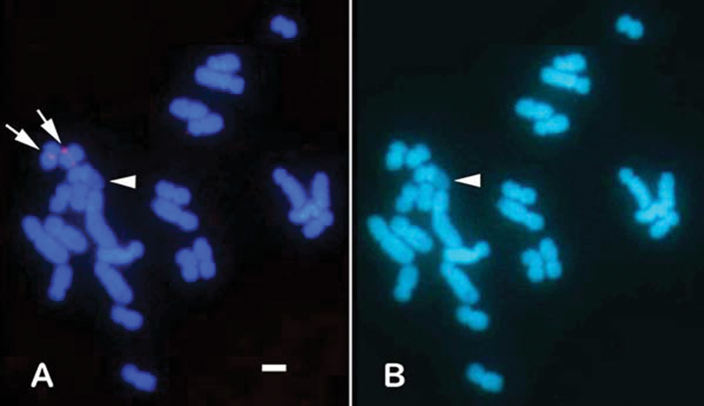
FISH image by MA-13C. FISH red signals by MA-13C (**A**; arrows) and DAPI staining (**B**). HSRs in **A** and **B** were easily identified due to pale portions (arrowheads). Note that two chromosomes 13 with MA-13C red signals (arrows) in **A** were discriminated from the chromosome 12 with an HSR. Scale bars: 2 μm.

**Figure 6.**
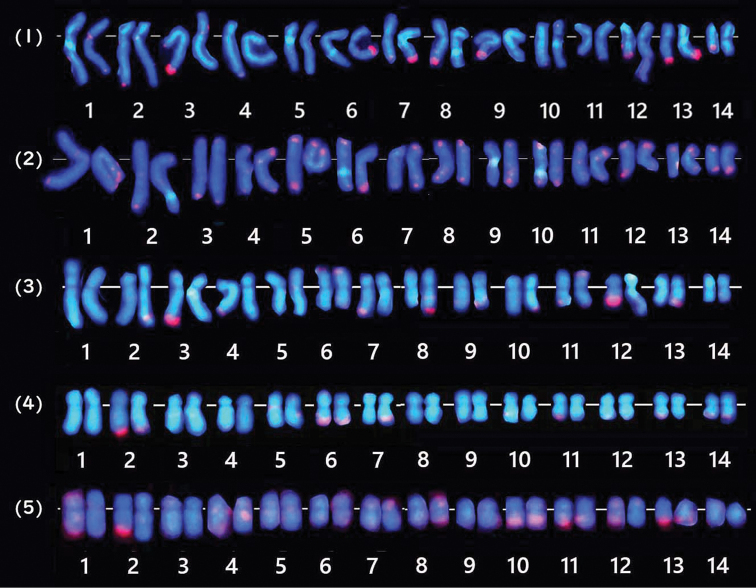
Five karyotyped FISH images using MA-TEL probe. Note that some chromosomes have signals on only one of the homologs and some other chromosomes show signals on both of the homologues at several telomere regions. Signal sizes and their distributions varied from a cell to cell.

**Figure 7.**
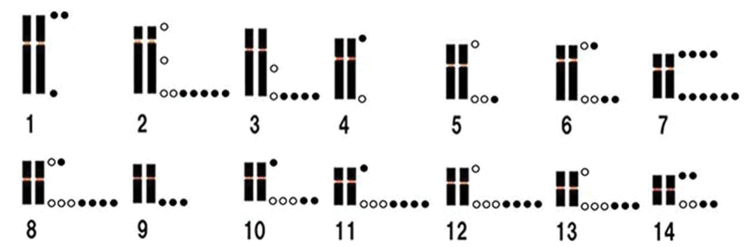
Diagram of the analyzed FISH signals which were observed on the chromosomes of seven metaphase spreads using the MA-TEL probe. Open circle: observed signals on both homologous chromosomes. Filled-dark circle: observed signals on only one chromosome of the homologous pair.

FISH analysis showed that the MA-H3 FISH marker (red signal) was detected near the centromere on the long arm of chromosome 1 (Fig. 4A). The MA-5S FISH marker hybridized on the telomere portion (green signal) of both homologous chromosomes 4 (Fig. 4B), indicating that *5S rRNA* gene is located on chromosome 4. The MA-18/28S FISH marker was mapped on the terminal portion of chromosome 12 (red signal, Fig. 4B). The extra-large red signal, which was conformable with the HSR, the pale portion as seen in the chromosomes counterstained with DAPI (Fig. 4B). This suggests that the *18S-28S rDNA* gene is located on the terminal portion of chromosome 12. The multiple copies of the *18S-28S* rDNA gene exist in the HSR of one chromosome 12 homologue. The MA-13C FISH marker generated bright, discrete signals specifically localized to the centromeric region of chromosome 13 (Fig. 4C), enabling clear differentiation of chromosome 13 from chromosome 12, which possesses the HSR (Fig. 5). In contrast, the MA-TEL FISH marker displayed signals along the telomere regions of several chromosomes (Figs 4D, 6). The distribution of telomeric signal varied among metaphase spreads, with signals observed on both homologous chromosomes, whereas in others, signals appeared on only one homologue (Figs 6, 7).

### ﻿Comparison of sequences of MA-H3, MA-5S (5S U2RNA), and MA-18/28S with those of other species

Sequence alignments of the cloned MA-H3, MA-5S, and MA-18/28S markers were compared to those of a variety of corals (Suppl. materials 6–8). The MA-H3 sequence was compared with homologous sequences from three other species (Suppl. material 6) and showed high similarity which was very much conserved. The MA-5S (*5S U2RNA*) sequence was compared with homologous sequences from three other species (Suppl. material 7). The MA-18/28S sequence was compared with homologous sequences from eight other species (Suppl. material 8).

## ﻿Discussion

### ﻿Cytogenetics

Molecular cytogenetic techniques, including conventional Giemsa staining and FISH, revealed a prominent HSR in more than 50% of *M.amakusensis* metaphase spreads. Generally, genetic amplification at the chromosomal level manifests as HSRs in tumor cells (Biedler and Spengler 1976). To date, seven molecular cytogenetic studies have been reported on seven stony corals, namely, *Echinophylliaaspera* (Taguchi et al. 2013), *Acroporasolitaryensis* (Taguchi et al. 2014), *Coelastreaaspera* (Taguchi et al. 2016), *Platygyracontorta* (Taguchi et al. 2017b), *A.pruinosa* (Taguchi et al. 2020; Vacarizas et al. 2021), *Favitespentagona* (Kawakami et al. 2022), and *Gonioporadjiboutiensis* (Vacarizas et al. 2023), all of these species exhibited HSRs, except *A.pruinosa*. However, conventional Giemsa staining does not consistently detect HSRs, likely because of the less distinct banding patterns in corals than in mammals, underscoring the need for an improved staining technique for coral cytogenetics. In this study, both Giemsa staining and FISH confirmed the presence of an HSR in *M.amakusensis*, which was localized at the terminal end of the long arm of chromosome 12. Furthermore, FISH analysis demonstrated that this HSR was derived from amplified rRNA-related genes. Although HSRs were observed in 50% of the metaphase spreads, the functional activity of over-amplified rDNA remains unclear. Nevertheless, the presence of an HSR appeared to be a characteristic feature of stony corals, except for *Acropora* spp.

### ﻿Genomic DNA Hybridization (GDH) using FISH

GDH is an effective cytogenetic method for detecting specific DNA repeats within a genome (Kallioniemi et al. 1992). In *M.amakusensis*, GDH analysis revealed that these repeats were predominantly located in telomeric regions, a characteristic that may serve as a distinguishing feature among stony corals. Similar telomeric FISH signal distributions have been observed in other species such as *Echinophylliaaspera* (Taguchi et al. 2013), *Trachyphylliageoffroyi* (Taguchi et al. 2017a), and *Gonioporalobata* (unpublished data). In contrast, in *Acropora* spp., these repeats are primarily observed in the centromeric regions. These specific chromosomal localizations of repeat DNAs may be helpful for the taxonomic classification of stony corals.

### ﻿Physical mapping by FISH

Multi-copy sequences (including genes) serve as valuable chromosomal markers for FISH techniques. Multi-copy sequences typically generate more prominent FISH signals than single-copy genes. Larger observable FISH signals resulting from a higher copy number are more easily detectable under a microscope. This feature is particularly important in stony corals, which have relatively small chromosomes compared to those of mammals (approximately half the size of human chromosomes). In this study, three genes (*histone H3*, *5S rRNA*, and *18S-28S rRNA*) were successfully mapped to their chromosomes, demonstrating their potential as effective FISH markers for chromosomal identification in karyotyping, as previously described (Taguchi et al. 2016; Kawakami et al. 2022). Importantly, these are the most common multi-copy genes used to compare sequence similarities among species for classification purposes (García-Souto et al. 2015; Suntronpong et al. 2017; Vacarizas et al. 2021). This is the first report of physical FISH mapping of *M.amakusensis* using MA-H3, MA-5S, and MA-18/28S probes. These three markers will help with the karyotyping of *M.amakusensis* by enabling discrimination among at least 3 out of 14 chromosome pairs. During screening with a combination of primers targeting *EF1-α* and *5S rRNA* genes, two additional probes were unexpectedly developed, namely MA-13C and MA-TEL. FISH signals were detected at the centromere of chromosome 13 using MA-13C and at the telomere regions of several chromosomes using MA-TEL. The MA-TEL probes highlighted several telomere regions on several chromosomes, revealing distinct signals on both the short and long arms at variable positions, suggesting the presence of one of the interspersed repeated sequences and non-coding DNA elements (Fowler et al. 1988; Taguchi et al. 2013). Notably, FISH signals from specific gene probes are generally observed in the same portions of homologous chromosomes. However, the MA-TEL produced variable signal patterns between metaphase spreads and sometimes marked only one homolog of a chromosome pair, likely due to differences in the number of MA-TEL-specific sequences present in each telomere region. The telomere regions of *M.amakusensis* may be unstable due to highly repeated sequences, such as HSRs (Taguchi et al. 2013, 2016, 2017a, b), resulting in variable signal distribution and sizes.

### ﻿Cloning and Sequence analysis

Five FISH markers (MA-H3, MA-5S, MA-18/28S, MA-13C, and MA-TEL) were successfully cloned and sequenced. Sequence homology analysis using NCBI (https://blast.ncbi.nlm.nih.gov/Blast.cgi?PROGRAM=blastn) showed that MA-H3 was part of the *M.amakusensis histone H3* gene (Suppl. materials 1, 6) and was more conserved than the other probes. MA-5S corresponded to the *5S rRNA* gene and matched *5S U2RNA*, with hits on various chromosomes of the *Echinoporahorrida* genome and predicted *5S rRNA* sequences of *Pocilloporaverrucosa*. The low query cover suggested that these were partial matches to highly conserved regions and that this probe was related to the *5S rRNA* gene (Suppl. materials 2, 7). This is the first report of the *U2RNA* of *5S rRNA* found in *M.amakusensis.* The MA-18/28S FISH marker matched the sequence of the original species *M.amakusensis*, it was determined to be a part of the *18S-28S rRNA* gene (Suppl. materials 3, 8). Although MA-13C was found only in the *Echinoporahorrida* genome and on chromosomes 2 and 16, the high number of hits likely reflects repetitive sequences within these genomic regions. Interestingly, the MA-TEL FISH marker showed no significant similarities with sequences from other corals. Since the *EF1-α* primer was used to construct this MA-TEL probe, the resulting PCR product may be related to this gene, but further study is needed as no database match was found.

Globally, there are more than 800 species of stony corals, but their classification remains unresolved due to conventional morphological traits (detailed skeletal morphology) and DNA sequence homology analyses (ribosomal DNA and mitochondrial DNA) have led to marked confusion (Fukami et al. 2004; Suzuki and Fukami 2012; Ng et al. 2019). Cytogenetics offers a complementary method for classification based on chromosomal characteristics. The advent of molecular cytogenetics has helped to differentiate coral species based on their genetic loci and syntenic relationships. Although several cytogenetic studies on Cnidaria (Anthozoa, Hydrozoa, and Scleractinia), stony coral cytogenetics (chromosome studies) can provide valuable insights into evolutionary relationships (Kenyon 1997; Flot et al. 2006; Taguchi et al. 2017a). Molecular cytogenetics is expected to bridge the gap between morphological and molecular approaches in coral taxonomy. In this study, detailed molecular cytogenetic characteristics of *M.amakusensis* were determined, and five FISH markers were cloned and sequenced to facilitate karyotype analysis. These findings contribute important information for the future classification of scleractinian corals.

In conclusion, the molecular cytogenetics analysis of scleractinian corals provides significant information for the classification of corals and even other invertebrates using FISH techniques. FISH significantly extends the capabilities of conventional cytogenetic analysis by rapidly generating multiple probes, which enables a detailed examination of previously uncharacterized chromosomal heterochromatin. In this study, we successfully performed chromosomal analysis using molecular cytogenetic techniques. Specifically, we mapped five FISH markers, MA-H3, MA-5S, MA-18/28S, MA-13C, and MA-TEL, to the chromosomes of *M.amakusensis* and subsequently cloned and sequenced them. Our findings demonstrated that these probes not only facilitate precise chromosome identification through distinct FISH signals but also offer valuable insights for clarifying coral classification based on sequence traits. These new findings will motivate further surveys of the cytogenetic features of other stony corals and help resolve taxonomic ambiguities in stony corals.

## ﻿Authors’ contributions

TT and SK: designed experiments. ABB, MI, HS, TM, and TT: performed experiments. ABB, MI, YN, SEM, and IJNB: organized the figures and tables and drafted the manuscript.TM: facilitated the sampling and identification of the specimens. TT, AT, and SK: supervised the research and corrected the manuscript. All authors read, discussed, and approved the final version of the manuscript.

## Supplementary materials

Supplementary material 1 MA-H3 (Histone H3: *M.amakusensis*) 282 bp Data type docx https://binary.pensoft.net/file/1390271This dataset is made available under the Open Database License (http://opendatacommons.org/licenses/odbl/1.0/). The Open Database License (ODbL) is a license agreement intended to allow users to freely share, modify, and use this Dataset while maintaining this same freedom for others, provided that the original source and author(s) are credited.Analyn B. Baldove, Masumi Ito, Takahiro Taguchi, Takuma Mezaki, Hiroumi Saito, Sam Edward Manalili, Yuji Namura, Ivy Jamela Nieves-Brutas, Akira Tominaga, Satoshi Kubota

Supplementary material 2 MA-5S (5S U2RNA: *M.amakusensis*) 920 bp Data type docx https://binary.pensoft.net/file/1390272This dataset is made available under the Open Database License (http://opendatacommons.org/licenses/odbl/1.0/). The Open Database License (ODbL) is a license agreement intended to allow users to freely share, modify, and use this Dataset while maintaining this same freedom for others, provided that the original source and author(s) are credited.Analyn B. Baldove, Masumi Ito, Takahiro Taguchi, Takuma Mezaki, Hiroumi Saito, Sam Edward Manalili, Yuji Namura, Ivy Jamela Nieves-Brutas, Akira Tominaga, Satoshi Kubota

Supplementary material 3 MA-18/28S (18/28S rRNA gene: *M.amakusensis*) 1258 bp Data type docx https://binary.pensoft.net/file/1390273This dataset is made available under the Open Database License (http://opendatacommons.org/licenses/odbl/1.0/). The Open Database License (ODbL) is a license agreement intended to allow users to freely share, modify, and use this Dataset while maintaining this same freedom for others, provided that the original source and author(s) are credited.Analyn B. Baldove, Masumi Ito, Takahiro Taguchi, Takuma Mezaki, Hiroumi Saito, Sam Edward Manalili, Yuji Namura, Ivy Jamela Nieves-Brutas, Akira Tominaga, Satoshi Kubota

Supplementary material 4 MA-13C (centromere: *M.amakusensis*) 111 bp Data type docx https://binary.pensoft.net/file/1390274This dataset is made available under the Open Database License (http://opendatacommons.org/licenses/odbl/1.0/). The Open Database License (ODbL) is a license agreement intended to allow users to freely share, modify, and use this Dataset while maintaining this same freedom for others, provided that the original source and author(s) are credited.Analyn B. Baldove, Masumi Ito, Takahiro Taguchi, Takuma Mezaki, Hiroumi Saito, Sam Edward Manalili, Yuji Namura, Ivy Jamela Nieves-Brutas, Akira Tominaga, Satoshi Kubota

Supplementary material 5 MA-TEL (Multi-sites on telomeres: *M.amakusensis*) 149 bp Data type docx https://binary.pensoft.net/file/1390275This dataset is made available under the Open Database License (http://opendatacommons.org/licenses/odbl/1.0/). The Open Database License (ODbL) is a license agreement intended to allow users to freely share, modify, and use this Dataset while maintaining this same freedom for others, provided that the original source and author(s) are credited.Analyn B. Baldove, Masumi Ito, Takahiro Taguchi, Takuma Mezaki, Hiroumi Saito, Sam Edward Manalili, Yuji Namura, Ivy Jamela Nieves-Brutas, Akira Tominaga, Satoshi Kubota

Supplementary material 6 Histone H3 gene Data type xlsx https://binary.pensoft.net/file/1390276This dataset is made available under the Open Database License (http://opendatacommons.org/licenses/odbl/1.0/). The Open Database License (ODbL) is a license agreement intended to allow users to freely share, modify, and use this Dataset while maintaining this same freedom for others, provided that the original source and author(s) are credited.Analyn B. Baldove, Masumi Ito, Takahiro Taguchi, Takuma Mezaki, Hiroumi Saito, Sam Edward Manalili, Yuji Namura, Ivy Jamela Nieves-Brutas, Akira Tominaga, Satoshi Kubota

Supplementary material 7 5S ribosomal RNA gene Data type xlsx https://binary.pensoft.net/file/1390277This dataset is made available under the Open Database License (http://opendatacommons.org/licenses/odbl/1.0/). The Open Database License (ODbL) is a license agreement intended to allow users to freely share, modify, and use this Dataset while maintaining this same freedom for others, provided that the original source and author(s) are credited.Analyn B. Baldove, Masumi Ito, Takahiro Taguchi, Takuma Mezaki, Hiroumi Saito, Sam Edward Manalili, Yuji Namura, Ivy Jamela Nieves-Brutas, Akira Tominaga, Satoshi Kubota

Supplementary material 8 18S ribosomal RNA gene Data type xlsx https://binary.pensoft.net/file/1390278This dataset is made available under the Open Database License (http://opendatacommons.org/licenses/odbl/1.0/). The Open Database License (ODbL) is a license agreement intended to allow users to freely share, modify, and use this Dataset while maintaining this same freedom for others, provided that the original source and author(s) are credited.Analyn B. Baldove, Masumi Ito, Takahiro Taguchi, Takuma Mezaki, Hiroumi Saito, Sam Edward Manalili, Yuji Namura, Ivy Jamela Nieves-Brutas, Akira Tominaga, Satoshi Kubota
