# Supplementary material for: ﻿Molecular cytogenetic study on the scleractinian coral Micromussaamakusensis (Veron, 1990) (Hexacorallia, Anthozoa, Cnidaria): isolation of five fluorescence in situ hybridization markers
Source: Comp Cytogenet. 2025 Aug 7;19:135–54. doi: 10.3897/compcytogen.19.157310 (PMC12355185; doi:10.3897/compcytogen.19.157310)
Supplement: Supplementary material 1 — MA-H3 (Histone H3: M.amakusensis) 282 bp [file comparative_cytogenetics-19-135_article-157310__-s001.docx]

| **TCACCAACTC GCTCAGCATA ACTTTGAAAG CGCAGGTCCG TTTTGAAATC** | **50** |
| --- | --- |
| **CTGTGCAATT TCTCGCACAA GACGCTGAAA GGGCAGCTTG CGGATGAGTA** | **100** |
| **GTTCTGTAGA TTTCTGATAA CGGCGAATCT CTCGAAGAGC AACTGTTCCG** | **150** |
| **GGTCTGTAAC GATGAGGCTT CTTGACACCA CCTGTAGCAG GAGCGCTCTT** | **200** |
| **CCGAGCTGCC TTTGTAGCTA ACTGCTTACG AGGAGCTTTT CCACCAGTTG** | **250** |
| **ATTTTCGAGC TGTCTGCTTG GTACGAGCCA TA** | **300** |

**Suppl. Fig. 1**

**MA-H3 (Histone H3: *M. amakusensis*) 282bp**
