# Supplementary material for: ﻿Molecular cytogenetic study on the scleractinian coral Micromussaamakusensis (Veron, 1990) (Hexacorallia, Anthozoa, Cnidaria): isolation of five fluorescence in situ hybridization markers
Source: Comp Cytogenet. 2025 Aug 7;19:135–54. doi: 10.3897/compcytogen.19.157310 (PMC12355185; doi:10.3897/compcytogen.19.157310)
Supplement: Supplementary material 2 — MA-5S (5S U2RNA: M.amakusensis) 920 bp [file comparative_cytogenetics-19-135_article-157310__-s002.docx]

**Suppl. Fig. 2**

**MA-5S (5S U2RNA: *M. amakusensis*) 920bp**

| **CTTCCATGAT CGGACGAGAA CCGGTGCTTT CCCTGGGGTA GGTCGTAGA** | **50** |
| --- | --- |
| **CAAGAGAATG AGATGAAAAT TTAGTGTTTT AACGGTCCGG TAAAACAATA** | **100** |
| **ATATGAACCA GGAATCTTTT AGCAGGCATT TCTTGACGTT TGTACAAGTT** | **150** |
| **TCATGCACGT ACAGAGCGAA CAATGATAAC GTAGCCGTTG CTTGTTGTCC** | **200** |
| **GTTTCTGTAA CGCTTTCTCA GTCACGCAGT CCAGTCATTA CCAGGGGGCA** | **250** |
| **AAAGAAATGG CCTCGTTTGG GAGGAGAAAA AGGGTGTTGA ATTACAGCCA** | **300** |
| **GCCCTGGAGG GACCTTATGA AGATTCTGCC GCTTTTTTGG GGACTAATGC** | **350** |
| **GGTGGGACAG GTGCCTCTAT GTCGGCAACT TGTTCCATAG CGTCCTCCTA** | **400** |
| **CAGTCGGCTC CAGGCCTCTG ATGTCAAGAA CAATGGTGTC AGCGGACAAC** | **450** |
| **ATATTGACCA CCACATCCGA AAGAGGGAAC CCCTGGACGA CCCTCCTAGA** | **500** |
| **GGAGGGAACC TTCGGTATCA CTTCTCGGCC TTTTGGCTAA GATCAAGAGG** | **550** |
| **TCTGAGTCTG TTTTTTTCCC GCCCTTCTTT TTGATTGGTG CTTTCGGAGT** | **600** |
| **TCCCAAGATG CCCTCCGCGC CGCGGTCTGG CGCCACTCCC GATCATGGAT** | **650** |
| **GTAACGTAGT TGTACAAGAT TTCCTTGCTG CCGATAATTC CTCGTTGAGT** | **700** |
| **TCTTCTTCTC GGTCCTCTGA GGTCCAAAGT GGTGATCCAG CAAGTGTACC** | **750** |
| **ATCCAATGCT GTAGATTCTG CAGGGCCTTC GGGCCCTGAA GCCGCCTCAA** | **800** |
| **GACCCGATGT TTCGTCCCAA GTTGTTTACT CCGATGTAGT ACGTGGATCT** | **850** |
| **ATCAACTCGC AACCTGTAGC TTATGTTCCC GATGAGCAGA ACGAATTGCC** | **900** |
| **TTCTCGTCCG ATCACGGAAG** | **950** |
