# Supplementary material for: ﻿Molecular cytogenetic study on the scleractinian coral Micromussaamakusensis (Veron, 1990) (Hexacorallia, Anthozoa, Cnidaria): isolation of five fluorescence in situ hybridization markers
Source: Comp Cytogenet. 2025 Aug 7;19:135–54. doi: 10.3897/compcytogen.19.157310 (PMC12355185; doi:10.3897/compcytogen.19.157310)
Supplement: Supplementary material 3 — MA-18/28S (18/28S rRNA gene: M.amakusensis) 1258 bp [file comparative_cytogenetics-19-135_article-157310__-s003.docx]

**Suppl. Fig. 3**

**MA-18/28S (18/28S rRNA gene: *M. amakusensis*) 1258bp**

| **GTACCCTTTT GTACACACCG CCCGTCGCTA CTACCGTTGA ATGGTTTAGT** | **50** |
| --- | --- |
| **GAGGCCTCCT GACTGGCGCC GATGCTCTGG CAACAGAGCG CCGGATGCCG** | **100** |
| **GAAAGTTGGT CAAACTTGAT CATTTAGAGG GAGTAAAAGT CGTAACAAGG** | **150** |
| **TTTCCGTAGG TGAACCTGCG GAAGGATCAT TACCGATACA AGGAGGCCCA** | **200** |
| **ACAACGACGA CTTTTTCGTC GTTTGCCCTG TGAACTGTAC TCAATCATCA** | **250** |
| **TTTAGGGGTC GGCCGTCCAG GCCGTTAAAT GGCGATCGGA CCAGGAATGG** | **300** |
| **GCCGAGTCGC CGGCCCCGGC AGTGTTTTTT TCGAACATTT ACGTGTCGAT** | **350** |
| **GGTCTGATGG TATCTATCGG TCCCCTGGCC GATAGGTCAA GAAAAAGAAA** | **400** |
| **TAGAGTGAGA CAACTTGTGG CGGTGGATCT CTTGGCTCGC GCGTCGATGA** | **450** |
| **AGAACGCAGC CTGCTGCGAT AAGCAGTGTG AATTGCAGAA TTCGGTGAAT** | **500** |
| **CATCGAACCT TTGAACGCAA ATGGCGCCCT TGGGTTGTCC CAGGAGCATG** | **550** |
| **TCCTGTTCTG AGTGTCATGA AATGCAAATG ATCCGCAGCA TCGCTTTTGG** | **600** |
| **CTTTGCTTTG CGGCGTTGAG GCGTCACGGC CCAACCCAAC AGGGCGGCCG** | **650** |
| **TGTCCCTTGA AAGGCAGAGA GGAGACTCGC TGCTGTGCAA AGCGGAGGCC** | **700** |
| **AAAATTCCTT CAGACCTTCG CCAGGCACAA TGCAATGCCA GTCGCCAAAA** | **750** |
| **AGAGGCGAAA CACCATTCTA TTGACCCTCA GATCAGGCAA GGCTACCCGC** | **800** |
| **TGAATTTAAG CATATTAATA AGCGGAGGAA AAGAAACTAA CAAGGATTCC** | **850** |
| **CTCAGTAACG GCGAGTGAAG CGGGAAGAGC TCAAATTTGA AATCTCCGAT** | **900** |
| **GCTTGCATCG GCGAATTGTA GTTGCGAGAA GCACTTTCTA GGCGGATCGC** | **950** |
| **TGGCGCCTAA GTTGCTTGGA ACGGCACGTC GCAGAGGGTG ACAACCCCGT** | **1000** |
| **CCGTGGCGCC ATCGGCCGCC GACGATGTGC TTTCGAAGAG TCGGGTTGTT** | **1050** |
| **TGGGAATGCA GCCCAAAATG GGTGGTAAAC TCCATCTGAA GCTAAATACT** | **1100** |
| **GGCGCGAGAC CGATAGCGAA CAAGTACCGC GAGGGAAAGA TGAAAAGAAC** | **1150** |
| **TTTGAAAAGA GAGTTAAAAA GTACGTGAAA CCGTCGCAAG GGAAACGAAT** | **1200** |
| **GGACTCAGCA ATGCGCCGTT TTGAGATTCA GCGACGGGCG GTGTGTACAA** | **1250** |
| **AGGGTACC** |  |
