# Supplementary material for: ﻿Molecular cytogenetic study on the scleractinian coral Micromussaamakusensis (Veron, 1990) (Hexacorallia, Anthozoa, Cnidaria): isolation of five fluorescence in situ hybridization markers
Source: Comp Cytogenet. 2025 Aug 7;19:135–54. doi: 10.3897/compcytogen.19.157310 (PMC12355185; doi:10.3897/compcytogen.19.157310)
Supplement: Supplementary material 4 — MA-13C (centromere: M.amakusensis) 111 bp [file comparative_cytogenetics-19-135_article-157310__-s004.docx]

**Suppl. Fig.4**

**MA-13C (centromere: *M. amakusensis*) 111bp**

| **ATTTACAAAT GTGGTGGTAT CGCCCTCTCG GTAAAAAAGG TCTATTACAC** | **50** |
| --- | --- |
| **AACTTTCAGC TTGTTTCTTT CGTCTACGAC CACACCACAG GAAACACACC** | **100** |
| **CGTTCTCGTC A** | **150** |
