# Supplementary material for: ﻿Molecular cytogenetic study on the scleractinian coral Micromussaamakusensis (Veron, 1990) (Hexacorallia, Anthozoa, Cnidaria): isolation of five fluorescence in situ hybridization markers
Source: Comp Cytogenet. 2025 Aug 7;19:135–54. doi: 10.3897/compcytogen.19.157310 (PMC12355185; doi:10.3897/compcytogen.19.157310)
Supplement: Supplementary material 5 — MA-TEL (Multi-sites on telomeres: M.amakusensis) 149 bp [file comparative_cytogenetics-19-135_article-157310__-s005.docx]

**Suppl. Fig. 5**

**MA-TEL (Multi-sites on telomeres: *M. amakusensis*) 149bp**

| **ATTTACAAAT GTGGTGGTAT CGGCAGAATA TCGGTAAAGT ATCGATAAGG** | **50** |
| --- | --- |
| **TATCGGCCGA GTGTCGATGA GACTAAGACC GTATCGGTCG ACACACACAT** | **100** |
| **CCGTCGATAT ATCGGCTGAT GCTCGGCTGA CACCCGTTCT CGTCCGATC** | **150** |
